# ANP and BNP Responses to Dehydration in the One-Humped Camel and Effects of Blocking the Renin-Angiotensin System

**DOI:** 10.1371/journal.pone.0057806

**Published:** 2013-03-13

**Authors:** Abdu Adem, Mahmoud Al Haj, Sheela Benedict, Javed Yasin, Nicolas Nagelkerke, Fred Nyberg, Tim G. Yandle, Chris M. Frampton, Lynley K. Lewis, M. Gary Nicholls, Elsadig Kazzam

**Affiliations:** 1 Departments of Pharmacology, Faculty of Medicine and Health Sciences, UAE University, Al Ain, United Arab Emirates; 2 Internal Medicine, Faculty of Medicine and Health Sciences, UAE University, Al Ain, United Arab Emirates; 3 Community Medicine, Faculty of Medicine and Health Sciences, UAE University, Al Ain, United Arab Emirates; 4 Department of Pharmaceutical Biosciences, Uppsala University, Uppsala, Sweden; 5 Department of Medicine, University of Otago - Christchurch, Christchurch Hospital, Christchurch, New Zealand; John Hunter Hospital, Australia

## Abstract

The objectives of this study were to investigate and compare the responses of atrial natriuretic peptide (ANP) and B-type natriuretic peptide (BNP) in the circulation of hydrated, dehydrated, and dehydrated losartan - treated camels; and to document the cardiac storage form of B-type natriuretic peptide in the camel heart. Eighteen male camels were used in the study: control or hydrated camels (n = 6), dehydrated camels (n = 6) and dehydrated losartan-treated camels (n = 6) which were dehydrated and received the angiotensin II (Ang II) AT-1 receptor blocker, losartan, at a dose of 5 mg/kg body weight intravenously for 20 days. Control animals were supplied with feed and water ad-libitum while both dehydrated and dehydrated-losartan treated groups were supplied with feed ad-libitum but no water for 20 days. Compared with time-matched controls, dehydrated camels exhibited a significant decrease in plasma levels of both ANP and BNP. Losartan-treated camels also exhibited a significant decline in ANP and BNP levels across 20 days of dehydration but the changes were not different from those seen with dehydration alone. Size exclusion high performance liquid chromatography of extracts of camel heart indicated that proB-type natriuretic peptide is the storage form of the peptide.

We conclude first, that dehydration in the camel induces vigorous decrements in circulating levels of ANP and BNP; second, blockade of the renin-angiotensin system has little or no modulatory effect on the ANP and BNP responses to dehydration; third, proB-type natriuretic peptide is the storage form of this hormone in the heart of the one-humped camel.

## Introduction

The one-humped camel (*Camelus dromedarius*), widely distributed in the Gulf countries, is well able to survive water deprivation for long periods without ill effects. Indeed, camels can tolerate a loss of water corresponding to 30% of body weight whereas other mammals may die from circulatory failure when water loss involves a much smaller percentage of body weight [1].

The maintenance of fluid, electrolyte and circulatory homeostasis during dehydration is likely to be dependant, in part, upon changes in circulating levels of hormones with known effects on sodium and water balance. Whereas the effects of dehydration on some of these hormones are well documented in certain species, this is not so for the camel where responses in circulating levels of the cardiac natriuretic peptides in particular are unknown. This is somewhat surprising given that these peptides are known to have actions which serve to maintain sodium and water balance and, in particular, to prevent circulatory overload through potent effects on the kidney (natriuresis and diuresis), the central nervous system (inhibition of thirst, salt appetite and antidiuretic hormone secretion) and the adrenal gland (inhibition of aldosterone secretion) [2]. Although interactions between the renin-angiotensin system and the cardiac natriuretic peptides are well documented under some circumstances and in some species [3], no such information is available across dehydration in the camel. Accordingly, we studied the effects in camels of dehydration on plasma levels of atrial natriuretic peptide (ANP) and B-type natriuretic peptide (BNP) and investigated whether blockade of angiotensin II (Ang II) type 1 receptors with losartan altered the natriuretic peptide response. Finally, we sought to establish the molecular form of BNP within the camel heart. This study was planned on the basis of three underlying hypotheses: first, that dehydration would induce a decline in plasma levels of ANP and BNP; second, that blockade of ANG II type 1 receptors would enhance the fall in ANP and BNP levels; and third, that the dominant form of BNP in the camel heart would be in a large molecular weight form consistent with proBNP.

## Materials and Methods

As described elsewhere [4], 18 male camels, aged 3–4 years and weighing 290–348 kg were studied. They were kept under shade in a corral during the summer months of June and July when maximum daily temperatures varied between 40 and 48°C. They were divided into three groups: control camels, 6 in number, were allowed free access to feed and water during the 20 day study. Twelve camels, studied at the same time as control animals, were denied water access but given food ad-lib for 20 days: 6 of these 12 dehydrated camels were given the Ang II type 1 (AT_1_) receptor blocker, losartan (Merck, USA), 5 mg/Kg body weight daily by injection into the external jugular vein. All camels were maintained on hay for the first week of the experiment and freshly cut green grass for the remainder of the study. Blood was collected from an external jugular vein between 08:00 and 10:00 hours two days before the start of the experiment (baseline) and again on day 20 of dehydration and control. Samples were collected for the measurement of circulating hormone levels, into K3-EDTA vacutainers placed on ice, centrifuged at +4°C within one hour and the plasma stored as aliquots at −80°C until analyzed. Plasma was couriered on dry ice to the Endolab in Christchurch New Zealand for measurements of ANP and BNP. For initial, separate studies of plasma forms of immunoreactive ANP and BNP prior to assay of the samples across dehydration, blood was drawn from the external jugular vein of two healthy camels into tubes on ice containing EDTA, centrifuged at 4°C and the plasma stored at −80°C. For examination of cardiac immunoreactive BNP, hearts from 2 healthy camels with free access to feed and water were obtained immediately upon slaughter, placed on dry ice and stored at −80°C until transferred, along with the above plasma samples, on dry ice by courier to the Endolab in Christchurch, New Zealand where, again, they were stored at −80°C until analyses were carried out. Plasma samples were extracted on solid-phase C18 cartridges (Sep-Pak, Waters Corp., Milford, MA) as described previously [5] prior to radioimmunoassay (RIA) for ANP and BNP. The BNP assay employed an antiserum to porcine BNP-26 (Bachem cat number T-4195), porcine BNP-26 for standards and radiolabelled porcine BNP-26 as tracer. ANP was measured in the same extracts as BNP with a RIA employing a locally raised antiserum (R31) as previously described [6].

To characterize the form of BNP in camel heart, approximately 1 g of left or right atrial or ventricular tissue was diced, boiled for 5 minutes in 10 volumes of distilled water containing 0.1% Triton X-100, acidified to 1 M with acetic acid and homogenized for 2 minutes at 24,000 rpm (Ultra-Turrax T25, IKA). Following centrifugation, the extract was separated on a G2000SW size exclusion HPLC column (Toyo Soda, Tokyo, Japan), equilibrated with 0.1% TFA in 20% acetonitrile at 0.5 ml/minute and collected in 0.5 ml fractions into tubes containing 10 µl of 1% triton ×100. The fractions were dried, resuspended in assay buffer and measured by RIA. The column was calibrated with the following standards (molecular weight): recombinant human proBNP containing an additional methionine residue at its N-terminus (12,037), human aminoterminal proBNP (8,457), human BNP (3,464) and human Ang II (1,046).

Parallelism of extract dilutions with the RIA standard curve was performed on serial 2-fold dilutions of left and right atrial and ventricular tissue extracts. Plasma samples were extracted on Sep-Pak cartridges as above and reconstituted as concentrated solutions that were then serially diluted prior to RIA.

In order to determine whether the dose of losartan administered was sufficient to affect angiotensin type 1 receptors, liver tissue was obtained at slaughter from 3 camels in each of the three dehydration study groups. Measurement of Ang II receptor binding to camel liver tissue was determined using an Ang II binding assay [7]. The specific binding of ^125^I-Ang II (0.36 nM) was displaced by different concentrations of unlabelled Ang II (0.001 –100 nM) in the presence of 1 uM losartan (AT-1 receptor selective antagonist) or 1 uM PD-123319 (AT-2 receptor selective antagonist). K_i_ values were calculated from IC_50_ values using the Cheng and Prusoff equation: K_i_ = IC_50_/1+[L]/Kd. Results are presented as mean±SEM of three experiments each done in triplicate.

### Statistical Analysis

The recorded values for all groups were expressed as mean±SEM. Differences between the 3 groups of camels for ANP and BNP levels were determined by one-way ANOVA using SPSS version 19 with time as a repeated factor. Statistical significance was assumed at P<0.05

## Results

B–type natriuretic peptide (BNP) in camel plasma and heart

**Figure 1:**
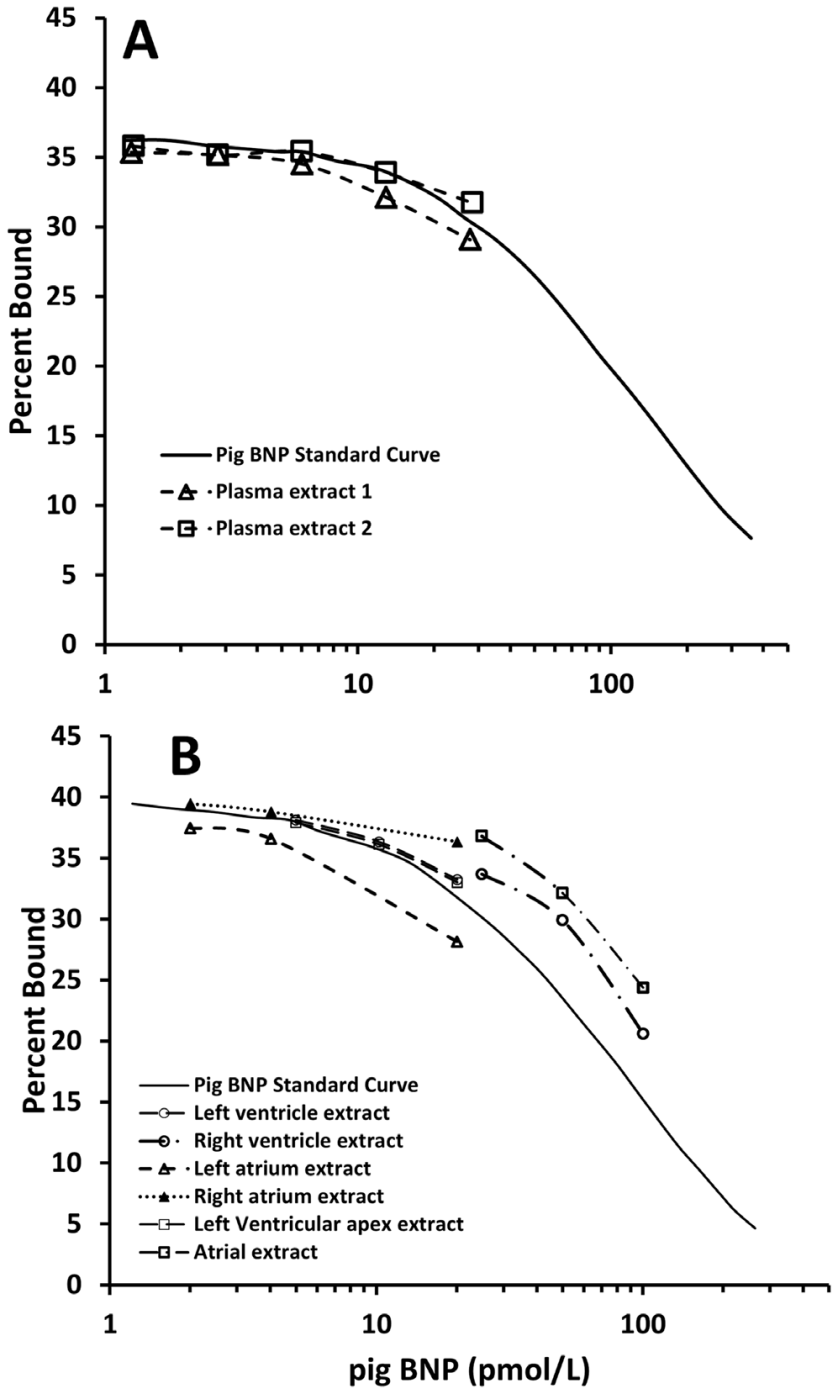
Dilutions of extracts of plasma (top panel) and heart (lower panel) from two camels in a radioimmunoassay utilizing standard porcine BNP1–32 and antiserum raised to porcine BNP.

Using a radioimmunoassay which utilized a polyclonal antibody raised to porcine BNP, camel plasma and extracts from left and right ventricle and left and right atria were diluted in parallel with standard porcine BNP1–32 (Figure 1). By contrast, there was no cross reactivity to standard human BNP1–32 and hence no parallelism, for camel heart extracts in a radioimmunoassay which used an antibody to human BNP1–32 (data not shown). Accordingly, plasma BNP levels from control, dehydrated and losartan-treated camels were measured by the radioimmunoassay utilizing an antibody to porcine BNP and porcine BNP standards.

**Figure 2:**
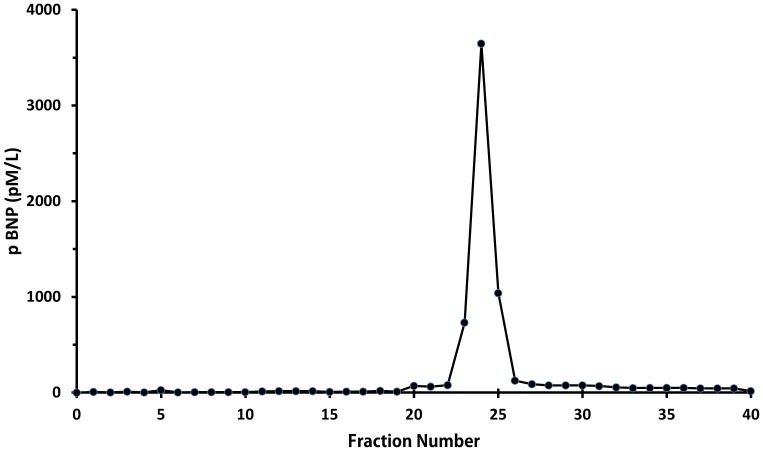
A single peak of immunoreactive BNP on size exclusion high performance liquid chromatography from an extract of camel atrial tissue using a radioimmunoassay with standard porcine BNP and antibody directed against porcine BNP. This peak corresponded exactly with that of large molecular weight proBNP, well separated from BNP1–32 and NT-proBNP, using the same technique for extracts of human heart (data not shown).

Size exclusion HPLC of extracts from camel atrial tissue demonstrated a single immunoreactive peak in fractions 24–26 (Figure 2) which eluted one fraction later than recombinant human proBNP standard but well ahead of synthetic human NTproBNP and BNP1–32 standards. This indicates that immunoreactive BNP in the camel heart is in the form of proBNP with an approximate molecular weight of 11,600 Da, and close to the theoretical molecular weight of camel proBNP (11,734 Da) calculated from the dromedary sequence provided by Osman et al and in Genebank:BAD21300.1 [8]. Further, it is evident that little or no immunoreactive BNP is stored as smaller molecular weight BNP or NT-proBNP.

### Effects of dehydration and losartan

Ang II receptors in the camel liver were exclusively of the AT-1 subtype. The displacement studies showed a marked increase (p<0.0001) and a significant decrease (p<0.0001) in the dehydrated (Ki = 0.06±0.005 nM) and losartan treated (Ki = 13.3±1.0 nM) camels respectively compared to controls (Ki = 0.98±0.001 nM).

**Figure 3:**
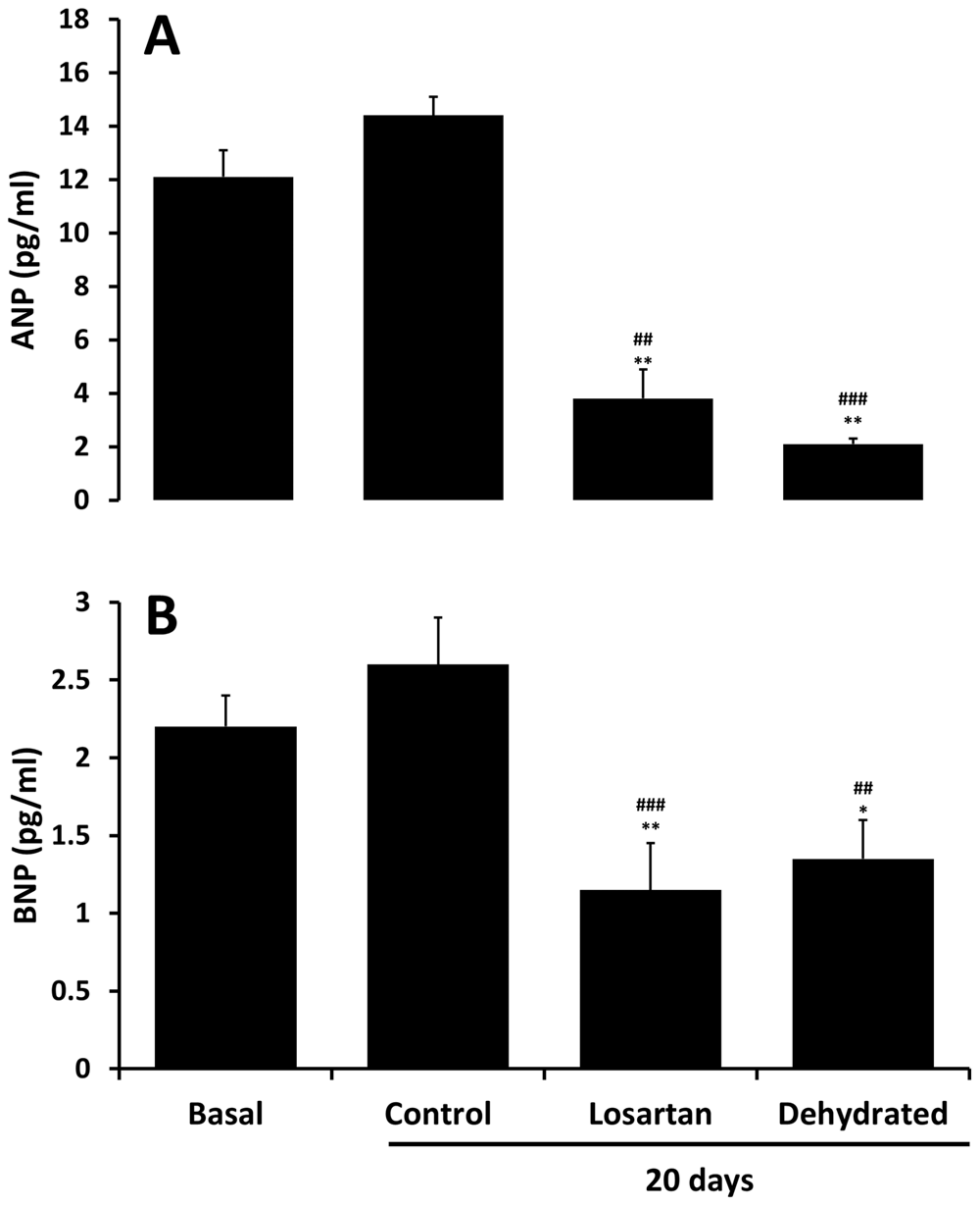
Plasma levels of ANP (top panel) and BNP (lower panel) in pmol/L at baseline and 20 days in control, dehydrated and losartan-treated/dehydrated camels. Data are shown as mean ±SEM. Basal versus losartan and dehydrated ^(*^P<0.05, ^**^P<0.01). Control versus losartan and dehydrated on day 20 (^##^P<0.01, ^###^P <0.001). To convert pg/ml to pmol/L for ANP multiplies by 0.32 and for BNP multiply by 0.29.

Plasma levels of ANP were approximately 6-fold higher than concomitant BNP levels at baseline (Figure 3). Whereas both ANP and BNP levels tended to rise over 20 days in control camels (NS) they decreased substantially and similarly across 20 days of dehydration with and without losartan administration (Figure 3).

## Discussion

The camel has an extraordinary capacity to survive water deprivation due in part, no doubt, to its ability to alter the production of hormones which are capable of altering sodium and water homeostasis. On the one hand are hormone systems including ADH, aldosterone, cortisol and the renin-angiotensin system which, under most circumstances, serve to retain water and/or sodium. On the other hand are the cardiac natriuretic peptides, ANP and BNP, which have potent diuretic and natriuretic actions. We earlier showed that 20 days of dehydration in the camel activates the renin-angiotensin system, induces substantial increments in serum sodium, creatinine, urea and arginine vasopressin (AVP) levels but had little effect on circulating aldosterone, and that blockade of Ang II type 1 receptors with losartan enhances the dehydration-induced fall in body weight and increments in serum creatinine and urea whilst reducing aldosterone and attenuating the rise in AVP [4]. Here, in the same camels, we have documented the effects of dehydration on circulating levels of the cardiac natriuretic peptides ANP and BNP. We also have determined the molecular form of BNP stored within the camel heart. Our novel observations are first, that dehydration across 20 days induces a substantial decline in circulating levels of ANP and BNP; second, that blockade of Ang II type 1 receptors does not further reduce levels of these two peptides across dehydration; and third, proBNP is the storage form of this hormone in the heart of the one-humped camel.

With regard to previous studies of the cardiac natriuretic peptides, 14 days of dehydration in camels was reported to have no effect on circulating levels of ANP [9]. In contrast, we observed that plasma ANP levels were substantially and statistically significant lower after 20 days of dehydration compared with time-matched controls. This response is unsurprising given that stretch of the cardiac chambers, generally considered the major stimulus to ANP release [10], would certainly have decreased across the dehydration period and, presumably, overcame any stimulatory action from increased activity of the renin-angiotensin system [10].

For measurements of plasma BNP, we first determined which antibody and standard should be used in our radioimmunoassay. This is vital since, by contrast with ANP, the amino acid structure of BNP is known to vary considerably across species and even amongst mammals. In the event, we found that both plasma and extracts of camel heart diluted in parallel with porcine BNP when using an antibody produced against porcine BNP. Our observations in this regard are congruent with an earlier study of camel natriuretic peptide cDNA which concluded that camel BNP has 94% identity with porcine BNP [8]. Our additional observation, using size-exclusion HPLC is that, as in many species including sheep [11], the dominant form of immunoreactive BNP in camel heart is a large molecular weight form consistent with the proBNP hormone - as we had hypothesized.

As for ANP and in accord with our hypothesis, dehydration resulted in a clear-cut decline in circulating levels of BNP. Again, as for ANP, we presume that any stimulatory action on BNP synthesis and release from activation of the renin-angiotensin system was overcome by reduced stretch of cardiac chambers during prolonged dehydration. Teleologically, these responses in plasma ANP and BNP levels would be seen as logical in being protective against excessive sodium and water loss. If, as would be expected, arterial pressure declined across dehydration in our camels, the natriuretic and diuretic actions of these peptides would be diminished [12], [13] thereby tending to further limit urinary loss of sodium and water. Nevertheless, caution is needed in this discussion since, to our awareness, there are no in-depth studies in camels regarding the regulation of cardiac natriuretic peptide release nor on their renal effects under conditions of water-depletion versus repletion.

In that the renin-angiotensin system interacts with the cardiac natriuretic peptides under some circumstances [2], [3], [10], we hypothesized that blockade of Ang II type 1 receptors might alter the responses of ANP and or BNP to dehydration. Our liver receptor study was carried out to determine whether the dose of losartan chosen was sufficient to block these receptors. The results indicate that there was a substantial effect on these receptors, at least in the liver. We cannot, of course, be certain that the receptor effects demonstrated in the liver occurred elsewhere and particularly in cardiac tissue. In any event losartan administration had no apparent effect to suppress further the effect of dehydration on plasma ANP and BNP concentrations.

In summary, 20 days of dehydration induced substantial suppression of plasma ANP and BNP concentrations. Blockade of Ang II type 1 receptors had little or no effect on the plasma ANP and BNP responses to dehydration. Finally, the storage form of B-type natriuretic peptide in the one-humped camel is large molecular weight proBNP.
